# Supplementary material for: Functional implications of dentition-based morphotypes in piscivorous fishes
Source: R Soc Open Sci. 2019 Sep 11;6(9):190040. doi: 10.1098/rsos.190040 (PMC6774978; doi:10.1098/rsos.190040)
Supplement: Supplemental Figures and Tables [file rsos190040supp1.docx]

**Supplemental Material**


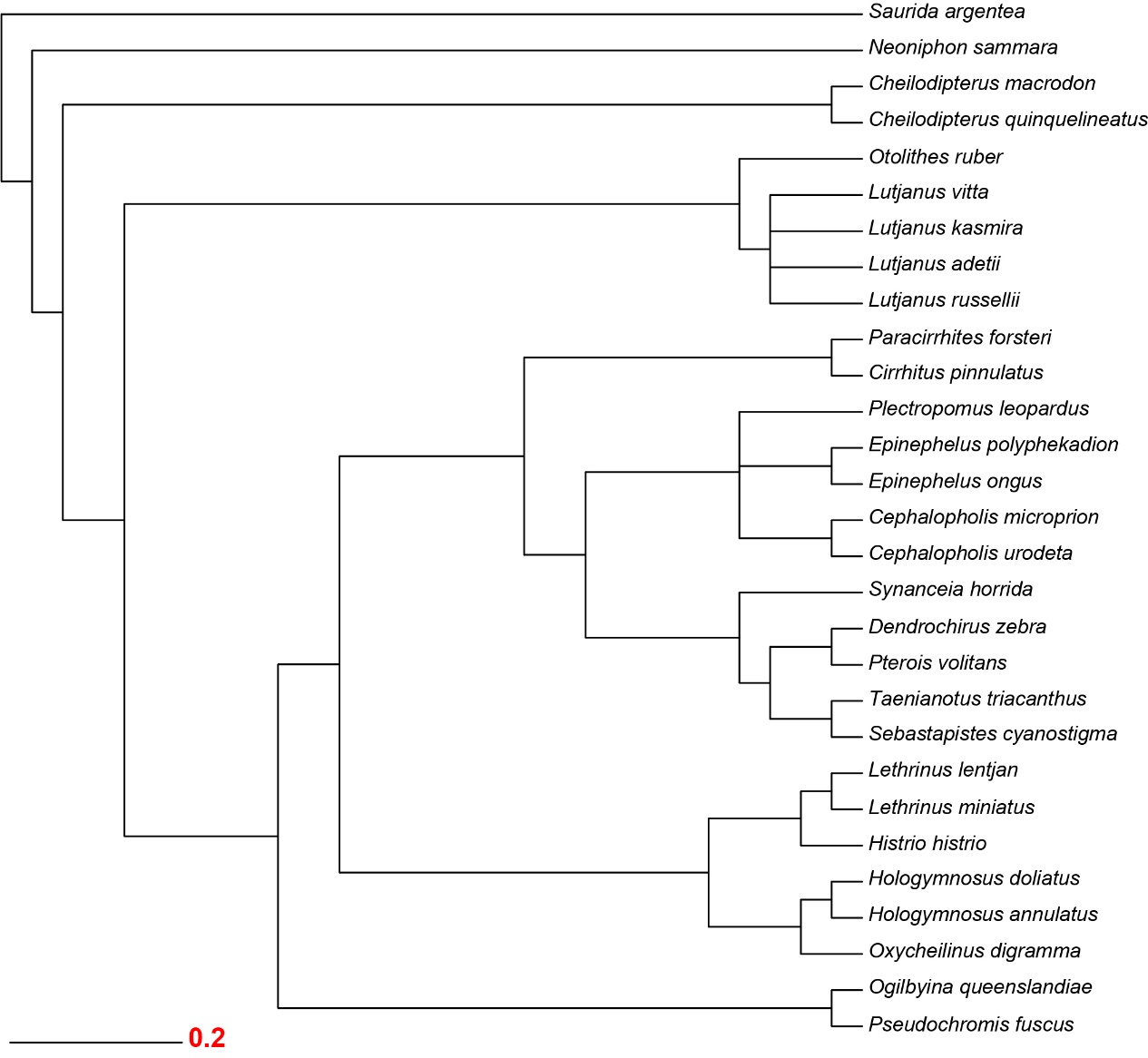


**Supplemental Fig. 1**. Phylogenetic tree used in our analyses


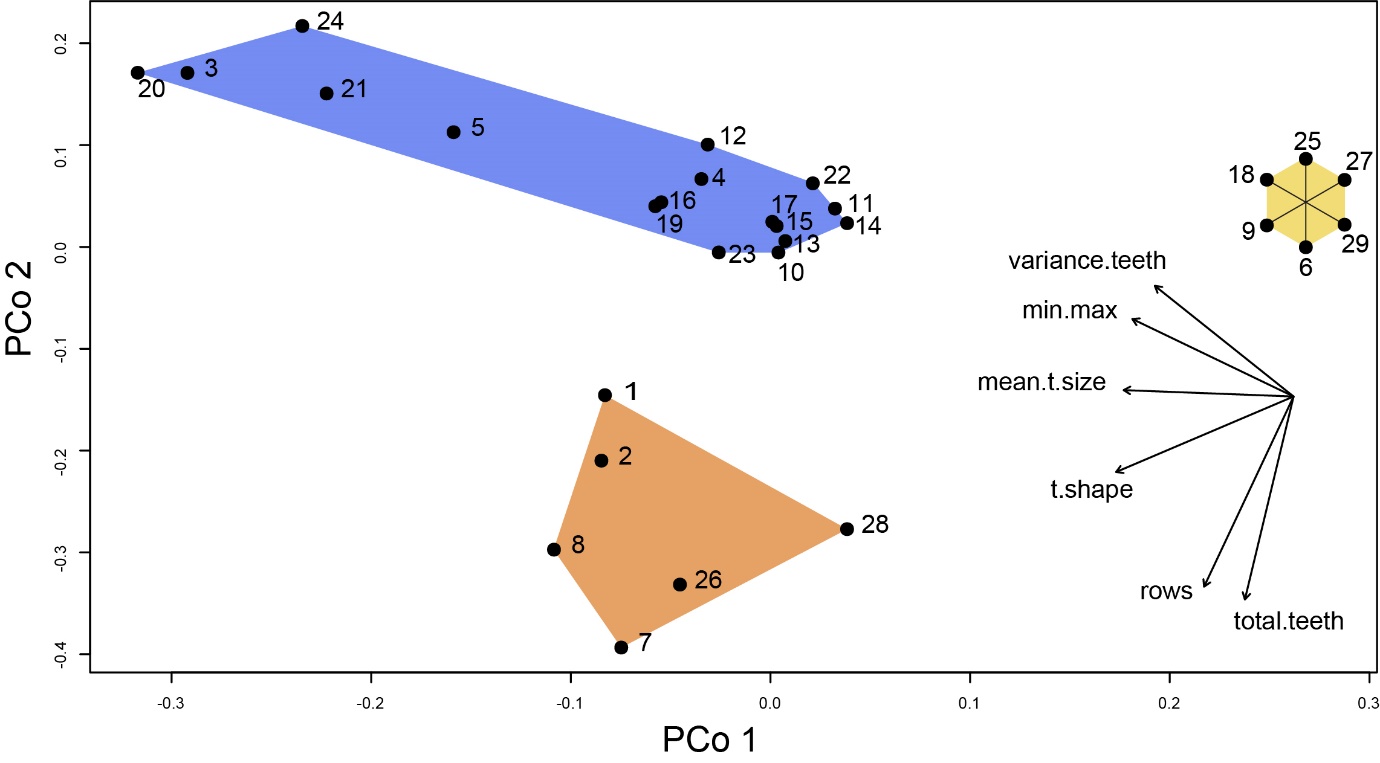


**Supplemental Fig. 2.** Principal Coordinate Analysis (PCoA). Species names: 1*-Cephalopholis microprion,* 2*-Cephalopholis urodeta,* 3*-Cheilodipterus macrodon,* 4*-Cheilodipterus quinquelineatus,* 5*-Cirrhitus pinnulatus,* 6*-Dendrochirus zebra,* 7*-Epinephelus ongus,* 8*-Epinephelus polyphekadion,* 9*-Histrio histrio,* 10*-Hologymnosus annulatus,* 11*-Hologymnosus doliatus,* 12*-Lethrinus lentjan,* 13*-Lethrinus miniatus,* 14*-Lutjanus adetii,* 15*-Lutjanus kasmira,* 16*-Lutjanus russellii,* 17*-Lutjanus vitta,* 18*-Neoniphon sammara,* 19*-Ogilbyina queenslandiae,* 20*-Otolithes ruber,* 21*-Oxycheilinus digramma,* 22*-Paracirrhites forsteri,* 23*-Plectropomus leopardus,* 24*-Pseudochromis fuscus,* 25*-Pterois volitans,* 26*-Saurida argentea,* 27*-Sebastapistes cyanostigma,* 28*-Synanceia horrida,* 29*-Taenianotus triacanthus.* For a detailed description of morphological traits, see Supplemental Table 1.


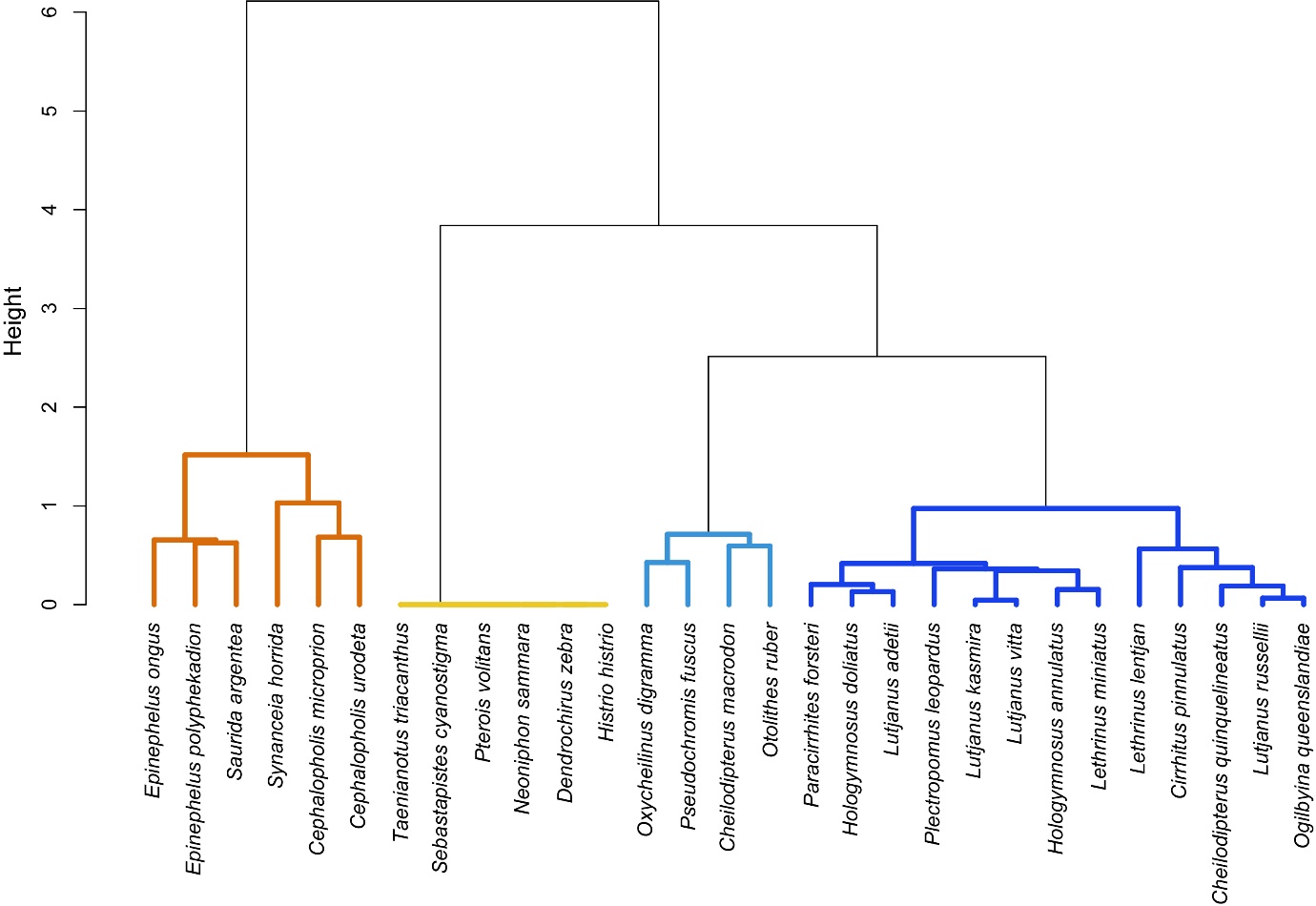


**Supplemental Fig. 3.** Hierarchical clustering analysis with SIMPROF analysis. Colours indicate significant clusters and are equivalent to colours used to indicate morphotypes in ordinations.


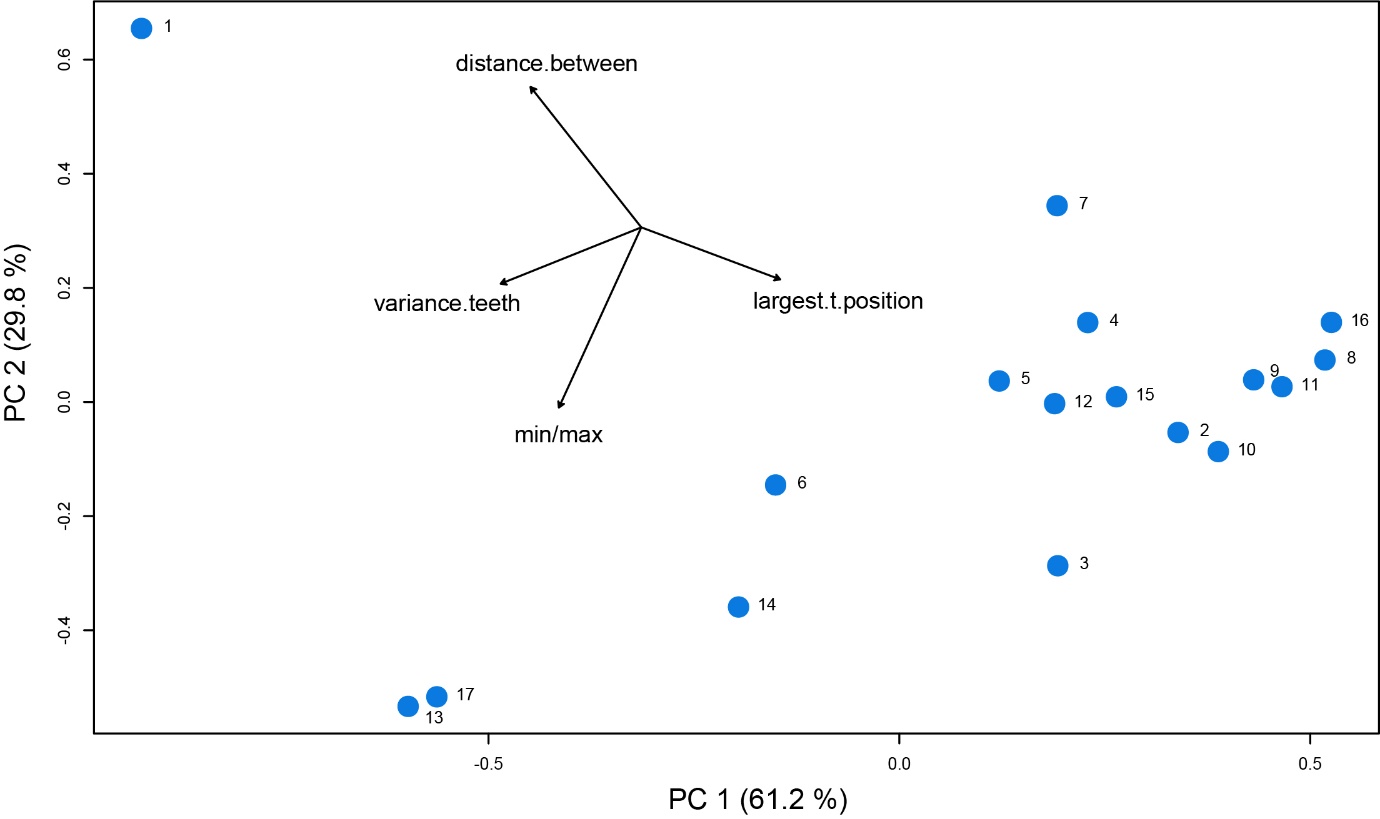


**Supplemental Fig. 4.** Macrodont-based Phylogenetic Principal Component Analysis (PPCA). Species names: 1*-Cheilodipterus macrodon,* 2*-Cheilodipterus quinquelineatus,* 3*-Cirrhitus pinnulatus,* 4*-Hologymnosus annulatus,* 5*-Hologymnosus doliatus,* 6*-Lethrinus lentjan,* 7*-Lethrinus miniatus,* 8*-Lutjanus adetii,* 9*-Lutjanus kasmira,* 10*-Lutjanus russellii,* 11*-Lutjanus vitta,* 12*-Ogilbyina queenslandiae,* 13*-Otolithes ruber,* 14*-Oxycheilinus digramma,* 15*-Paracirrhites forsteri,* 16*-Plectropomus leopardus,* 17*-Pseudochromis fuscus.* For a detailed description of traits used see Supplemental Table 1.


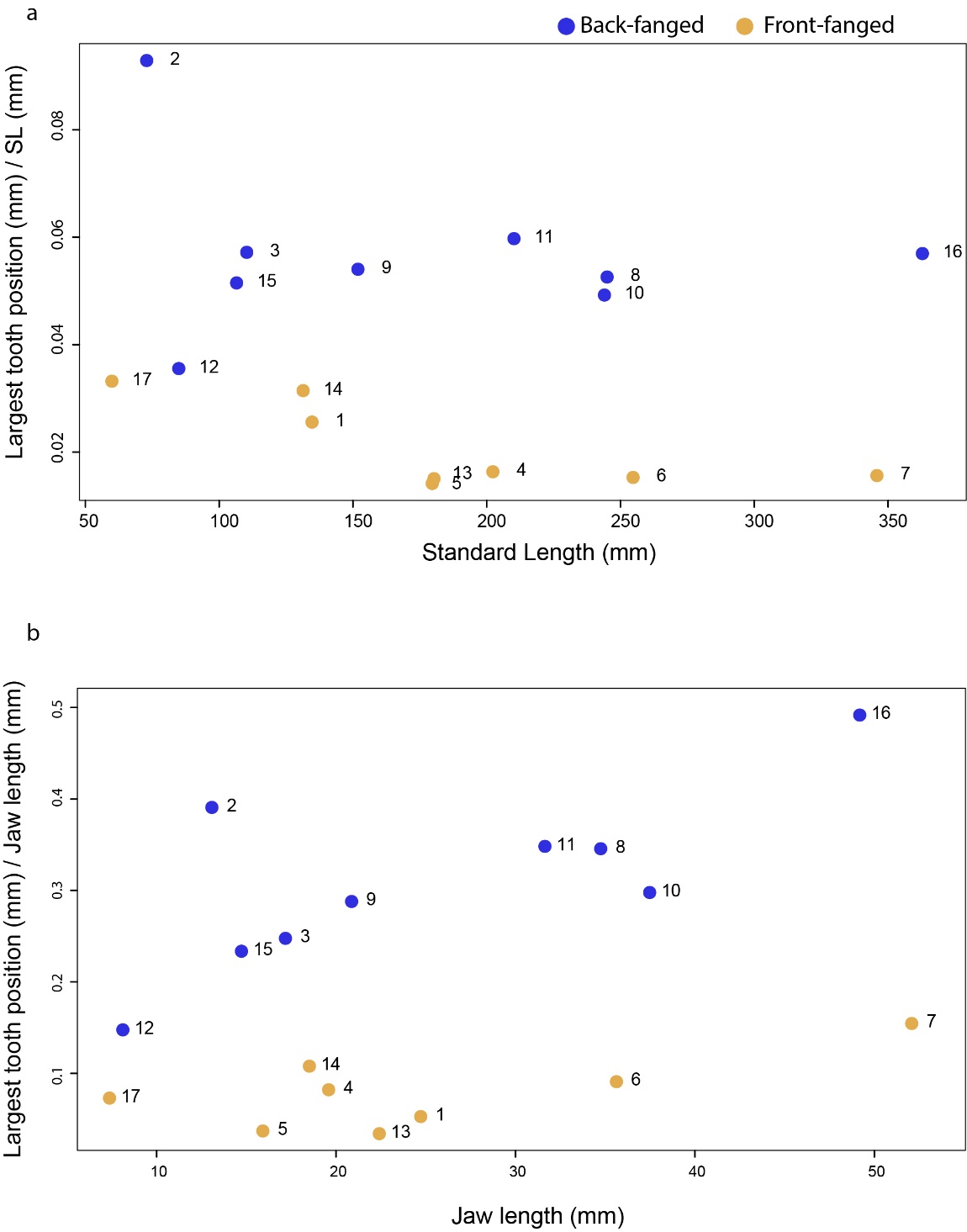


**Supplemental Fig. 5.** a) Position of largest tooth as distance from tip of the jaw, divided by body size (SL) to SL. b) Position of largest tooth as distance from tip of the jaw, divided by jaw length, to jaw length. Species names: 1*-Cheilodipterus macrodon,* 2*-Cheilodipterus quinquelineatus,* 3*-Cirrhitus pinnulatus,* 4*-Hologymnosus annulatus,* 5*-Hologymnosus doliatus,* 6*-Lethrinus lentjan,* 7*-Lethrinus miniatus,* 8*-Lutjanus adetii,* 9*-Lutjanus kasmira,* 10*-Lutjanus russellii,* 11*-Lutjanus vitta,* 12*-Ogilbyina queenslandiae,* 13*-Otolithes ruber,* 14*-Oxycheilinus digramma,* 15*-Paracirrhites forsteri,* 16*-Plectropomus leopardus,* 17*-Pseudochromis fuscus.* Colours represent respectively `back-fanged`(blue) and `front-fanged`(yellow) species.


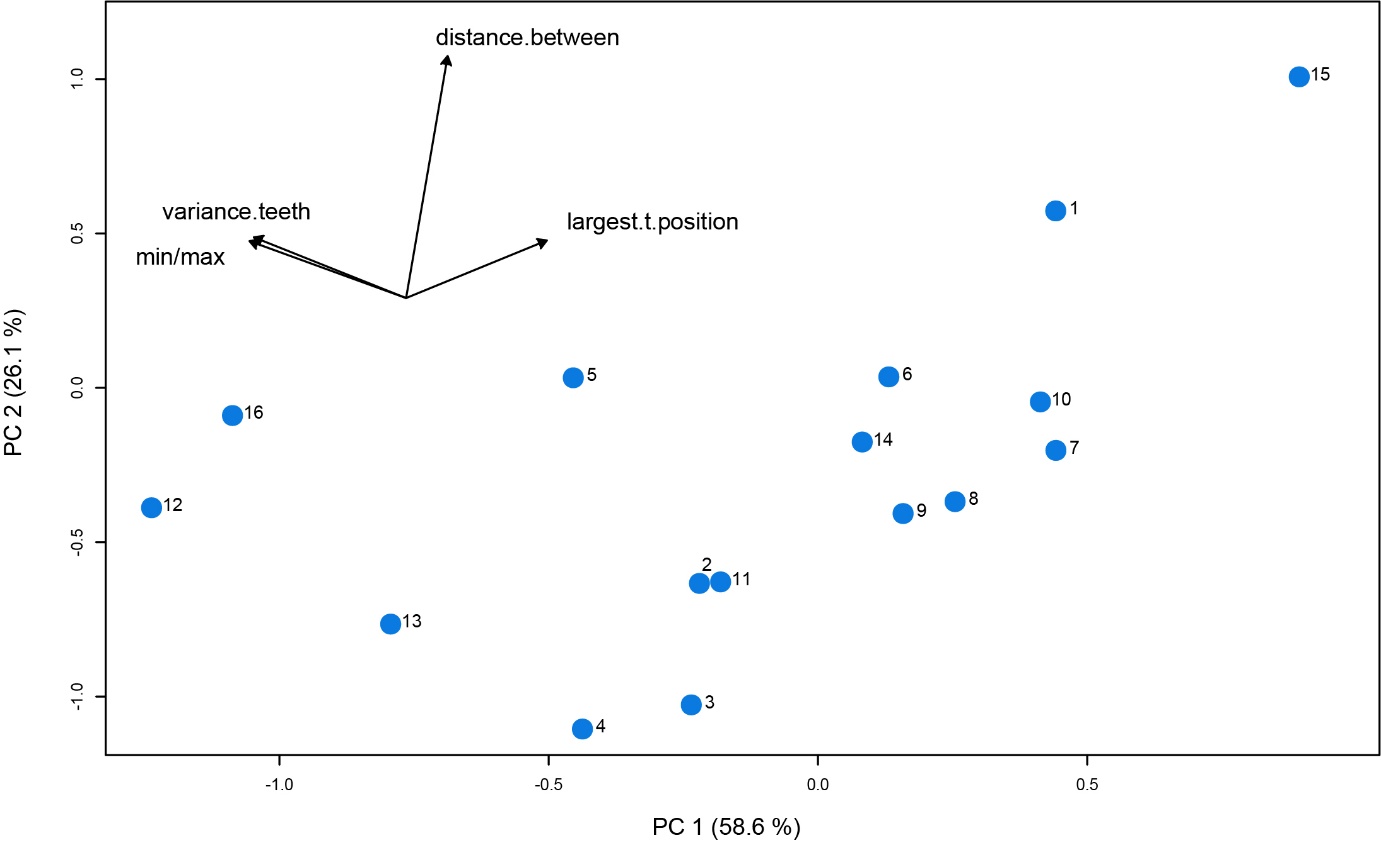


**Supplemental Fig. 6.** Macrodont Phylogenetic Principal Component Analysis (PPCA). Species names: 1*-Cheilodipterus quinquelineatus,* 2*-Cirrhitus pinnulatus,* 3*-Hologymnosus annulatus,* 4*-Hologymnosus doliatus,* 5*-Lethrinus lentjan,* 6*-Lethrinus miniatus,* 7*-Lutjanus adetii,* 8*-Lutjanus kasmira,* 9*-Lutjanus russellii,* 10*-Lutjanus vitta,* 11*-Ogilbyina queenslandiae,* 12*-Otolithes ruber,* 13*-Oxycheilinus digramma,* 14*-Paracirrhites forsteri,* 15*-Plectropomus leopardus,* 16*-Pseudochromis fuscus.*

**Supplemental Table 1.** Morphological traits used in our analyses. Analysis 1 indicates traits used in our morphotype analysis (Fig. 1), whereas Analysis 2 indicates traits used in our macrodont analysis (Fig. 3). Some of the morphological measurements were used to quantify some of the traits (e.g. jaw length used to quantify relative position of largest tooth).

| **Trait/Morphological meas.** | **Description** | **Analysis** |
| --- | --- | --- |
| Jaw length | length from anterior tip of anteriormost tooth, to the midpoint of the articular-quadrate articulation | 2 |
| tooth length (1-5) | tooth length from tip to the midpoint at the base | 1+2 |
| distance to jaw tip (1-5) | distance from midpoint at the base of tooth, to the anterior tip of the jaw. Used to calculate distance between teeth | 2 |
| largest tooth position | position of the largest tooth relative to jaw length (Out lever) | 2 |
| Variance in teeth size | variance in the tooth size of the 5 largest teeth of the left lower jaw. | 1+2 |
| Mean distance between teeth | Mean distance between the 5 largest teeth of the left lower jaw, relative to jaw length | 2 |
| tooth shape | maximum tooth width, measured at the base of the tooth. Used to calculate the ratio between tooth length / tooth width | 1 |
| min/max | ratio between the smallest (of the five largest teeth) and the largest tooth | 1+2 |
| total number of teeth | total number of teeth on left lower jaw | 1 |
| teeth rows | number of rows of teeth on left lower jaw | 1 |
| mean tooth size | mean length of the 5 largest teeth of the left lower jaw | 1 |

**Supplementary Table 2.** Functional feeding traits used in our analysis.

| **Trait** | **Description/Function** | **Refferences** |
| --- | --- | --- |
| Jaw protrusion | Increases the speed and grasping range of the bite | [1, 2 ] |
| Gape size | Influences maximum ingestible prey size | [3, 4] |
| Distance between teeth | More distance between teeth increases the ability to puncture flesh | [5, 6] |
| mouth shape | Influences suction feeding. More round = more efficient suction | [7] |
| jaw lever ratios (Lo & Li) | velocity vs. force potential | [8] |

**Supplementary Table 3.** Vector loadings from Phylogenetic Principal Component Analysis (PPCA).

| **Morphotype PPCA** | **PC1** | **PC2** |
| --- | --- | --- |
| variance teeth size | -0.6 | 0.664 |
| min/max | -0.457 | 0.304 |
| tooth shape | -0.913 | 0.109 |
| tooth abundance | -0.667 | -0.705 |
| rows | -0.715 | -0.664 |
| mean tooth size | -0.637 | 0.483 |
| **Functional PPCA** | **PC1** | **PC2** |
| Vert. Oral gape | -0.9 | 0.092 |
| Horiz. Oral gape | -0.874 | 0.453 |
| Protrusion | -0.256 | 0.181 |
| Mouth shape | 0.462 | -0.795 |
| Li/Lo | 0.816 | 0.546 |
| Lo/Li | -0.813 | -0.551 |
| **Macrodonts PPCA** | **PC1** | **PC2** |
| Largest tooth position | 0.819 | 0.224 |
| variance teeth size | -0.886 | 0.236 |
| min/max | -0.911 | -0.221 |
| distance between teeth | 0.242 | 0.942 |

**Supplementary Table 4.** Phylogenetic Least Squares (PGLS) models conducted on functional traits.

| **Trait (Response)** | **Morphotype (Explanatory)** | **AIC** | **BIC** | **logLik** | **Value** | **Std. Error** | **t-value** | **p-value** |
| --- | --- | --- | --- | --- | --- | --- | --- | --- |
| **Horizontal gape** |  | -66.69 | -61.22 | 37.345 |  |  |  |  |
|  | **Edentulate (Intercept)** |  |  |  | 0.378 | 0.075 | 5.075 | 0 |
|  | **Villiform** |  |  |  | -0.111 | 0.052 | -2.13 | 0.043 |
|  | **Macrodont** |  |  |  | -0.272 | 0.042 | -6.422 | 0 |
|  |  |  |  |  |  |  |  |  |
| **Vertical gape** |  | -97.338 | -91.869 | 52.669 |  |  |  |  |
|  | **Edentulate (Intercept)** |  |  |  | 0.324 | 0.044 | 7.384 | 0 |
|  | **Villiform** |  |  |  | -0.098 | 0.03 | -3.247 | 0.003 |
|  | **Macrodont** |  |  |  | -0.196 | 0.025 | -7.873 | 0 |
|  |  |  |  |  |  |  |  |  |
| **Jaw Protrusion** |  | 46.929 | 52.398 | -19.464 |  |  |  |  |
|  | **Edentulate (Intercept)** |  |  |  | 1.539 | 0.529 | 2.91 | 0.007 |
|  | **Villiform** |  |  |  | 0.464 | 0.366 | 1.265 | 0.217 |
|  | **Macrodont** |  |  |  | 0.092 | 0.3 | 0.307 | 0.761 |
|  |  |  |  |  |  |  |  |  |
| **Mouth shape** |  | 0.669 | 6.138 | 3.665 |  |  |  |  |
|  | **Edentulate (Intercept)** |  |  |  | 0.831 | 0.238 | 3.489 | 0.002 |
|  | **Villiform** |  |  |  | 0.057 | 0.165 | 0.349 | 0.73 |
|  | **Macrodont** |  |  |  | 0.221 | 0.135 | 1.634 | 0.114 |
|  |  |  |  |  |  |  |  |  |
| **Li/Lo** |  | -48.093 | -42.624 | 28.047 |  |  |  |  |
|  | **Edentulate (Intercept)** |  |  |  | 0.29 | 0.102 | 2.825 | 0.009 |
|  | **Villiform** |  |  |  | -0.016 | 0.071 | -0.221 | 0.826 |
|  | **Macrodont** |  |  |  | 0.12 | 0.058 | 2.056 | 0.05 |
|  |  |  |  |  |  |  |  |  |
| **Lo/Li** |  | 91.916 | 97.385 | -41.958 |  |  |  |  |
|  | **Edentulate (Intercept)** |  |  |  | 5.91 | 1.148 | 5.144 | 0 |
|  | **Villiform** |  |  |  | -0.417 | 0.796 | -0.524 | 0.604 |
|  | **Macrodont** |  |  |  | -2.622 | 0.653 | -4.013 | 0 |

**References**

[1] Lauder, G.V. & Liem, K.F. 1981 Prey capture by Luciocephalus pulcher: implications for models of jaw protrusion in teleost fishes. *Environmental Biology of Fishes* **6**, 257-268.

[2] Liem, K.F. 1967 A morphological study of Luciocephalus pulcher, with notes on gular elements in other recent teleosts. *Journal of morphology* **121**, 103-133.

[3] Mihalitsis, M. & Bellwood, D.R. 2017 A morphological and functional basis for maximum prey size in piscivorous fishes. *PloS one* **12**, e0184679.

[4] Wainwright, P.C. & Richard, B.A. 1995 Predicting patterns of prey use from morphology of fishes. *Environmental Biology of Fishes* **44**, 97-113.

[5] Vogel, S. 2013 *Comparative biomechanics: life's physical world*, Princeton University Press.

[6] Whitenack, L.B., Simkins Jr, D.C. & Motta, P.J. 2011 Biology meets engineering: the structural mechanics of fossil and extant shark teeth. *Journal of morphology* **272**, 169-179.

[7] Lauder, G.V. 1979 Feeding mechanics in primitive teleosts and in the halecomorph fish Amia calva. *Journal of Zoology* **187**, 543-578.

[8] Wainwright, P.C. & Bellwood, D. 2002 Ecomorphology of Feeding in Coral Reef Fishes. In *Coral reef fishes: dynamics and diversity in a complex ecosystem* (ed. S. Peter), p. 33.
