# Supplementary material for: Functional implications of dentition-based morphotypes in piscivorous fishes
Source: R Soc Open Sci. 2019 Sep 11;6(9):190040. doi: 10.1098/rsos.190040 (PMC6774978; doi:10.1098/rsos.190040)
Supplement: R software code [file rsos190040supp3.docx]

Code

#written by Michalis Mihalitsis

#-------------------------------------------------------------------------------------

rm(list=ls()) #clears R workspace (helps prevent errors from old data)

#-------------------------------------------------------------------------------------

setwd('C://Users//Ejer//Desktop//PhD//Fish Teeth. Morphology and Function')

library(readxl)

Dataset=read_excel('Teeth Data.xlsx', sheet="raw.2")

#load packages

library(stats)

library(tidyr)

library(dplyr)

#-------------------------------------------------------------------------------------

Dataset = Dataset %>% select(c(`Family`,`Species`,`SL`,`MD`,`MW`,

`a.`,`b.`,`c.`,

`Protrusion (%SL)`:`Lo/Li`))%>%

mutate(Species=as.factor(Species),Family=as.factor(Family))%>%

arrange(Species) %>% group_by(Species,add = T)

Dataset = Dataset[!is.na(Dataset$tooth1),] #%>% na.omit

Dataset = Dataset[-c(1,3,18,19,58,59,65,66,67,69,72,73),]

Dataset = Dataset %>% select (-Family)

#==========================================================================

md.std = Dataset$MD/Dataset$SL

lm.md = lm(md.std~Dataset$SL)

mw.std = Dataset$MW/Dataset$SL

lm.mw= lm(mw.std~Dataset$SL)

a.std = Dataset$a./Dataset$SL

lm.a = lm(a.std~Dataset$SL)

a.res = resid(lm.a)

a.res = a.res + abs(min(a.res))+0.01

a.res = sqrt(a.res)

c.std = Dataset$c./Dataset$SL

lm.c = lm(c.std~Dataset$SL)

c.res = resid(lm.c)

c.res = c.res + abs(min(c.res))+0.01

c.res = sqrt(c.res)

lm.prot=lm(Dataset$`Protrusion (%SL)`~SL, data = Dataset)

lm.prot.res=resid(lm.prot)

lm.prot.res=lm.prot.res+abs(min(lm.prot.res))+0.01

lm.prot.res = sqrt(lm.prot.res)

dent.std=Dataset$`dentary l`/Dataset$SL

lm.dent = lm(dent.std~SL, data = Dataset)

jaw.std=Dataset$`jaw l`/Dataset$SL

lm.jaw = lm(jaw.std~SL, data = Dataset)

tooth1.std=Dataset$tooth1/Dataset$SL

lm.tooth1 = lm(tooth1.std~SL, data = Dataset)

d1.std=Dataset$d1/Dataset$SL

lm.d1 = lm(d1.std~SL, data = Dataset)

tooth2.std=Dataset$tooth2/Dataset$SL

lm.tooth2 = lm(tooth2.std~SL, data = Dataset)

d2.std=Dataset$d2/Dataset$SL

lm.d2 = lm(d2.std~SL, data = Dataset)

tooth3.std=Dataset$tooth3/Dataset$SL

lm.tooth3 = lm(tooth3.std~SL, data = Dataset)

d3.std=Dataset$d3/Dataset$SL

lm.d3 = lm(d3.std~SL, data = Dataset)

tooth4.std=Dataset$tooth4/Dataset$SL

lm.tooth4 = lm(tooth4.std~SL, data = Dataset)

d4.std=Dataset$d4/Dataset$SL

lm.d4 = lm(d4.std~SL, data = Dataset)

tooth5.std=Dataset$tooth5/Dataset$SL

lm.tooth5 = lm(tooth5.std~SL, data = Dataset)

d5.std=Dataset$d5/Dataset$SL

lm.d5 = lm(d5.std~SL, data = Dataset)

lm.d5.res=resid(lm.d5)

lm.d5.res=lm.d5.res+abs(min(lm.d5.res))+0.01

bd.1.2.std = Dataset$bd.1.2/Dataset$SL

lm.bd.1.2 = lm(bd.1.2.std~SL, data = Dataset)

bd.2.3.std = Dataset$bd.2.3/Dataset$SL

lm.bd.2.3 = lm(bd.2.3.std~SL, data = Dataset)

bd.3.4.std = Dataset$bd.3.4/Dataset$SL

lm.bd.3.4 = lm(bd.3.4.std~SL, data = Dataset)

bd.4.5.std = Dataset$bd.4.5/Dataset$SL

lm.bd.4.5 = lm(bd.4.5.std~SL, data = Dataset)

largest.t.pos.std = Dataset$largest.t.pos/Dataset$SL

lm.largest.t.pos = lm(largest.t.pos.std~SL, data=Dataset)

lm.largest.t.pos.res = resid(lm.largest.t.pos)

lm.largest.t.pos.res= lm.largest.t.pos.res+abs(min(lm.largest.t.pos.res))+0.01

l.t.width.std = Dataset$largest.t.width/Dataset$SL

lm.l.t.width = lm(l.t.width.std~SL, data = Dataset)

total.teeth.std = Dataset$`total teeth`/Dataset$SL

rows.std = Dataset$rows/Dataset$SL

#==============================================================================

Dataset2=data.frame(SL=Dataset$SL,Species=Dataset$Species,

a=a.std,c=c.std,prot=lm.prot.res, dent= dent.std,

jaw=jaw.std, t1=tooth1.std,d1=d1.std,

t2=tooth2.std,d2=d2.std,t3=tooth3.std,

d3=d3.std,t4=tooth4.std,d4=d4.std,

t5=tooth5.std,d5=lm.d5.res,bd.1.2=bd.1.2.std,

bd.2.3=bd.2.3.std,bd.3.4=bd.3.4.std,

bd.4.5=bd.4.5.std,l.t.width.std,lm.largest.t.pos.res,

total.teeth=Dataset$`total teeth`,rows=Dataset$rows,

'Li/Lo'=Dataset$`Li/Lo`,'Lo/Li'=Dataset$`Lo/Li`)

write.csv(Dataset2,"Dataset2.csv")

Dataset2 = Dataset2 %>% group_by(Species) %>%

summarise_all(mean,na.rm=T)%>% na.omit

#1)

largest.t.pos=Dataset2$lm.largest.t.pos.res

#which is the relative position of the largest tooth

#2)

t=Dataset2[,c('t1','t2','t3','t4','t5')] %>% as.data.frame()

min.value = function(x){min(x[x>0])}

min.tooth = apply(t,1,min.value)

min.max = min.tooth/apply(t,1,max)

min.max = 1-min.max

min.max = ifelse(min.max==-Inf, 0,min.max)

mean.t.size = apply(t,1,mean)

mean.t.size <- ifelse(is.na(mean.t.size), 0, mean.t.size)

#which is the ratio between the smallest and larggest teeth rel lengths

#3) Variances of: teeth size,distance to tip, distance between teeth

pos.tip = data.frame(Dataset2$d1,Dataset2$d2,Dataset2$d3,Dataset2$d4,

Dataset2$d5) %>% na.omit()

pos.bet = data.frame(Dataset2$bd.1.2,Dataset2$bd.2.3,Dataset2$bd.3.4,

Dataset2$bd.4.5)

pos.bet = replace(pos.bet,pos.bet<0,NA)

variance.dist.bet = apply(pos.bet,1,function(x) var(x[!is.na(x)]))

#making sure var is calculated only by non NA values

#-----------------------------------------------------

variance.teeth = apply(t,1,function(x) var(x[!is.na(x)]))

variance.teeth = replace(variance.teeth,is.na(variance.teeth),0)

variance.dist.tip = apply(pos.tip,1,var)

Dataset2= na.omit(Dataset2)

t1=Dataset[,c('Species','tooth1','tooth2','tooth3','tooth4','tooth5',

'largest.t.width')] %>%

as.data.frame() %>% group_by(Species) %>%

summarise_all(mean,na.rm=T)%>% na.omit

t.shape = apply(t1[c('tooth1','tooth2','tooth3','tooth4',

'tooth5')],1,max)/t1$largest.t.width

t.shape = replace(t.shape, is.nan(t.shape), 0)

data.ord = data.frame(#variance.dist.bet,variance.dist.tip,

#largest.t.pos,

variance.teeth,min.max,t.shape,total.teeth=Dataset2$total.teeth,

rows=Dataset2$rows,mean.t.size)

rownames(data.ord)= Dataset2$Species

write.csv(data.ord,'data.ord.csv')

#Getting a Phylogenetic Tree from Open Tree of Life

rm(list=ls()) #clears R workspace (helps prevent errors from old data)

#-------------------------------------------------------------------------------------

setwd('C://Users//Ejer//Desktop//PhD//Fish Teeth. Morphology and Function')

library(readxl)

library(rotl)

library(phytools)

library(ape)

library(vegan)

library(dplyr)

data.ord = read.csv('Dataset2.csv')

data.ord = data.ord %>% group_by(Species) %>%

summarise_all(mean,na.rm=T)%>% na.omit

taxon.search= tnrs_match_names(names = as.character(data.ord$Species),

context_name = "Vertebrates")

#which matches names from your dataset to trees online. If TRUE,

#it means name found is not exactly the same, so check spelling or species

#might have new name. Results from this gives the matches.

#------------

data.ord$ott.names=taxon.search$unique_name

data.ord$ott.id=taxon.search$ott_id

#which adds 'Tree of Life' names and IDs to your dataset

tr = tol_induced_subtree(ott_ids=data.ord$ott.id)

#which gives an Error!

#however some of the species are not available in the 'Tree of Life'so

#we remove them

data.tree = data.ord[!data.ord$ott.id %in% c('3633703'),]

#where %in% says match the ott.id vector with the c() vector, and all of

#that is extracted from the 'factors'dataframe.

#Then we retry to make the tree.

tr = tol_induced_subtree(ott_ids=data.tree$ott.id)

#but our data and tree tip labels have different names (underscores and id

#labels)

tr$tip.label = strip_ott_ids(tr$tip.label, remove_underscores=TRUE)

tr$tip.label %in% data.tree$ott.names

tr = compute.brlen(tr,method = "Grafen")

write.tree(tr,file = "tr.tre")

#which matches tree tip labels with species names in our dataset.

#===================NEXT WE ADD THE SPECIES REMOVED, MANUALLY====================

library(ape)

library(phytools)

tr=read.tree(file = "tr.tre")

tr= compute.brlen(tr,method = "Grafen")

#and then we use bind.tip. First we add Aulostomus ch. (ott.id:231974)

tr2=bind.tip(tr,"Ogilbyina queenslandiae",where= 1,

position= 0.5*tr$edge.length[which(tr$edge[,2]==1)])

plot.phylo(tr2, type = 'phylogram',use.edge.length = T,

cex = 0.6,no.margin = T,root.edge = T)

nodelabels(cex=0.5,frame = 'none')

#============Checking for misspelled names between dataset and tree===============

tr2$tip.label= gsub("_"," ",tr2$tip.label)

Misspelled = tr2$tip.label[which(!tr2$tip.label %in% data.ord$Species)]

#data.ord$Species = gsub("Oxycheilinus digrammus","Oxycheilinus digramma",

# data.ord$Species)

tr2$tip.label = gsub("Lates calcarifer -species in Deuterostomia","Lates calcarifer",

tr2$tip.label)

tr2$tip.label = gsub("Alectis indica","Alectis indicus",

tr2$tip.label)

Misspelled = tr2$tip.label[which(!tr2$tip.label %in% data.ord$Species)]

Misspelled

#=================================================================================

#Now you need to do the same for the 'Envar' data frame

#Envar$Species = gsub("Diagramma pictum","Diagramma picta",Envar$Species)

#Envar$Species = gsub("Epinephelus quoyannus","Epinephelus quoyanus", Envar$Species)

#Envar$Species = gsub("Oxycheilinus digrammus","Oxycheilinus digramma",Envar$Species)

#Envar$Species = gsub("Alectis indicus","Alectis indica", Envar$Species)

#Envar$Species = gsub("Carangoides caeruleopinnatus","Carangoides coeruleopinnatus",Envar$Species)

#Envar$Species = gsub("Psammoperca vaigiensis","Psammoperca waigiensis",Envar$Species)

#which renames the species in the dataset that don't match the tree tip labels.

rownames(data.ord) = data.ord$Species

tr = tr2 %>% compute.brlen(tr2, method = "Grafen", power = 1)

#which computes branch lengths for tree

plot.phylo(tr, type = 'phylogram',use.edge.length = T,cex = 0.6,

no.margin = T,root.edge = T,x.lim = NULL,y.lim =NULL)

nodelabels(cex=0.5,frame = 'none')

add.scale.bar(x=0.01,y=0.55, cex = 0.6, font = 2, col = "red")

write.tree(tr, file = "tr.tre")

#=============================PHYLOGENETIC PCA=================================

library(ape)

library(phytools)

library(vegan)

tr=read.tree(file='tr.tre')

tr$tip.label = gsub("_"," ",tr$tip.label)

data.ord.2 = read.csv('data.ord.csv')

rownames(data.ord.2) = data.ord.2$X

data.ord.2 = data.ord.2[,2:7]

Misspelled = tr$tip.label[which(!tr$tip.label %in% rownames(data.ord.2))]

rownames(data.ord.2)=gsub("Oxycheilinus digrammus","Oxycheilinus digramma",

rownames(data.ord.2))

Misspelled = tr$tip.label[which(!tr$tip.label %in% rownames(data.ord.2))]

Misspelled

phyl.pca=phyl.pca(tr,data.ord.2,method = "BM",mode = "corr")

Eigenvalues = diag(phyl.pca$Eval)

Total.var = sum(diag(phyl.pca$Eval))

PC1 = Eigenvalues[1]/Total.var

PC2 = Eigenvalues[2]/Total.var

PC3 = Eigenvalues[3]/Total.var

phyl.biplot = biplot(phyl.pca,var.axes=T,cex=0.8,xlabs=1:nrow(data.ord.2),

main="Phylogenetic PCA",

pch = 24, xlab = "PC 1", ylab = "PC 2")

pca.scr = phyl.pca$S

phy.pca.plot.1=plot(pca.scr[,1],pca.scr [,2],main="Phylogenetic PCA",

pch = 16,cex=1.8,

xlab = "PC 1 (44.7 %)", ylab = "PC 2 (26.6 %)")

text (pca.scr[,1] + 0.1,pca.scr [,2], labels = 1:nrow(data.ord.2),cex = 0.6)

library(clustsig)

library(plyr)

library(dendextend)

library(dendroextras)

library(factoextra)

library(NbClust)

pca.scr=as.matrix(pca.scr)

SIMP=simprof(data.ord.2,num.expected = 1000,

num.simulated = 999,method.cluster = "ward.D2",

method.distance = "euclidean",alpha = 0.01)

simp.groups = SIMP$significantclusters

par(oma=c(1,1,1,1))

par(mar=c(12,8,2,8)+0.1)

simprof.plot(SIMP,leafcolors = c('red','purple','blue','orange','green'),

plot=T,fill=T)

##############################################################################

#===========NOW FOR THE FUNCTIONAL ORDINATION============================

func.ord = read.csv('func.ord.csv')

rownames(func.ord)=func.ord$X

func.ord = func.ord[,2:7]

func.ord = log10(func.ord)

Envar1 = read.csv('Envar1.csv')

phyl.pca=phyl.pca(tr,func.ord,method = "BM",mode = "corr")

Eigenvalues = diag(phyl.pca$Eval)

Total.var = sum(diag(phyl.pca$Eval))

PC1 = Eigenvalues[1]/Total.var

PC2 = Eigenvalues[2]/Total.var

phyl.biplot = biplot(phyl.pca,var.axes=T,cex=0.8,xlabs=1:nrow(func.ord),

main="Phylogenetic PCA",

pch = 24, xlab = "PC 1", ylab = "PC 2")

pca.scr = phyl.pca$S

phy.pca.plot.1=plot(pca.scr[,1],pca.scr [,2],main="Phylogenetic PCA",

pch = 16,cex=1.8,

xlab = "PC 1 (49.5 %)", ylab = "PC 2 (28.8 %)")

text (pca.scr[,1] + 0.07,pca.scr [,2], labels = 1:nrow(func.ord),cex = 0.6)

yellow=c(rgb(214,174,62,max=255))

red=c(rgb(196,68,65,max=255))

blue=c(rgb(28,55,211,max=255))

con.hull.hab = ordihull(pca.scr, groups = Envar1$Type,

draw = 'polygon',

col=c(blue,red,yellow),border = NULL)

#=================PHYLOGENETIC PCA FOR MACRODONTS ONLY=================

tr=read.tree(file='tr.tre')

tr$tip.label = gsub("_"," ",tr$tip.label)

#Macrodonts=read.csv('Macrodont.csv')

#tips.to.cut = which(!tr$tip.label %in% Macrodonts$Species)

#tr = drop.tip(tr,tips.to.cut)

macs.ord = read.csv('data.macs2.csv')

rownames(macs.ord)=macs.ord$X

macs.ord = macs.ord[,2:7]

#removing C.macrodon

macs.ord = macs.ord[2:17,]

#removing lever ratios

macs.ord = macs.ord[,c(1,2,3,6)]

Envar1 = read.csv('Envar1.csv')

phyl.pca=phyl.pca(tr,macs.ord,method = "BM",mode = "corr")

Eigenvalues = diag(phyl.pca$Eval)

Total.var = sum(diag(phyl.pca$Eval))

PC1 = Eigenvalues[1]/Total.var

PC2 = Eigenvalues[2]/Total.var

phyl.biplot = biplot(phyl.pca,var.axes=T,cex=0.8,xlabs=1:nrow(macs.ord),

main="Phylogenetic PCA",

pch = 24, xlab = "PC 1", ylab = "PC 2")

pca.scr = phyl.pca$S

phy.pca.plot.1=plot(pca.scr[,1],pca.scr [,2],main="Phylogenetic PCA",

pch = 16,cex=1.8,

xlab = "PC 1 (55 %)", ylab = "PC 2 (26.1 %)")

text (pca.scr[,1] + 0.03,pca.scr [,2], labels = 1:nrow(macs.ord),cex = 0.6)

library(vegan)

library(ape)

d2=vegdist(macs.ord,method = "gower")

PCOA=pcoa(d2,correction = "none")

vecpcoa = PCOA$vectors[,1:2]

ord.plot.3=plot(vecpcoa[,1],vecpcoa [,2],

pch = 16,cex=1.8,

xlab = "PCo 1", ylab = "PCo 2")

text (vecpcoa[,1]+0.02,vecpcoa[,2], labels = 1:nrow(macs.ord),

cex = 0.6)

Code for macrodonts dataset

rm(list=ls()) #clears R workspace (helps prevent errors from old data)

#-------------------------------------------------------------------------------------

setwd('C://Users//Ejer//Desktop//PhD//Fish Teeth. Morphology and Function')

library(readxl)

#Dataset=read_excel('Teeth Data.xlsx', sheet="raw.2")

#load packages

library(stats)

library(tidyr)

library(dplyr)

Species = read.csv('Macrodont.csv')

Envar1=read.csv('Envar1.csv')

Envar1 = Envar1[Envar1$Species %in% Species$Species,]

Dataset2=read.csv('Dataset2.csv')

Dataset2 = Dataset2[Dataset2$Species %in% Species$Species,]

func.ord = read.csv('func.ord.csv')

Dataset2 = Dataset2 %>% group_by(Species) %>%

summarise_all(mean,na.rm=T)%>% na.omit

t=Dataset2[,c('t1','t2','t3','t4','t5')] %>% as.data.frame()

min.value = function(x){min(x[x>0])}

min.tooth = apply(t,1,min.value)

min.max = min.tooth/apply(t,1,max)

min.max = 1-min.max

min.max = ifelse(min.max==-Inf, 0,min.max)

#Variances of: teeth size,distance to tip, distance between teeth

raw = read_excel('Teeth Data.xlsx', sheet="raw.2")

raw = raw %>% select(c(`Family`,`Species`,`SL`,`MD`,`MW`,

`a.`,`b.`,`c.`,

`Protrusion (%SL)`:`Lo/Li`))%>%

mutate(Species=as.factor(Species),Family=as.factor(Family))%>%

arrange(Species) %>% group_by(Species,add = T)

raw = raw[which(raw$Species %in% Dataset2$Species),]

raw = raw[!is.na(raw$tooth1),] %>% summarise_all(mean,na.rm=F)

#----------------largest tooth relative position macs--------

l.t.rel = (raw$largest.t.pos/raw$`jaw l`)*100

l.t.rel = l.t.rel + raw$d1

t.pos.to.sl = Dataset2$lm.largest.t.pos.res

plot(t.pos.to.sl~raw$SL,pch=16)

text(raw$SL+10,t.pos.to.sl,labels = 1:nrow(raw))

t.pos.to.jl = raw$largest.t.pos/raw$`jaw l`

plot(t.pos.to.jl~raw$`jaw l`,pch=16)

text(raw$`jaw l`+1,t.pos.to.jl,labels = 1:nrow(raw))

#-----------------------------------------------------------

pos.bet = raw[c('jaw l','bd.1.2','bd.2.3','bd.3.4','bd.4.5')] %>% as.data.frame()

pos.bet = apply(pos.bet, 2, function(x) {ifelse(x < 0, abs(x), x)}) %>% as.data.frame()

pos.bet = apply(pos.bet, 2, function(x) {ifelse(x ==100, 0, x)}) %>% as.data.frame()

pos.bet = (pos.bet[,2:5]/pos.bet[,1]) *100

pos.bet = apply(pos.bet, 2, function(x) {ifelse(x == 0, 100, x)}) %>% as.data.frame()

pos.bet = apply(pos.bet, 1, mean)

#lm.pos.bet = lm(pos.bet~raw$SL)

#Ontogenetic effects not found significant

variance.dist.bet = apply(pos.bet,1,function(x) var(x[!is.na(x)]))

#making sure var is calculated only by non NA values

#-----------------------------------------------------

#first we need to account for some fish having less than 5 teeth (C.macrodon:3.67,1.89,2.52,1.98,0), since

#this influences variance calculation.

t = na_if(t,0)

variance.teeth = apply(t,1,function(x) var(x[!is.na(x)]))

#Now that variance calculations are correct, we can change back edentulates (NA) to zeros (0)

variance.teeth = replace(variance.teeth,is.na(variance.teeth),0)

variance.dist.tip = apply(pos.tip,1,var)

Dataset2= na.omit(Dataset2)

#tooth shape is a ratio, so not from Dataset2

#(which has both std values and residuals)

t1=Dataset2[,c('Species','t1','t2','t3','t4','t5',

'l.t.width.std')] %>%

as.data.frame() %>% group_by(Species) %>%

summarise_all(mean,na.rm=T)%>% na.omit

t.shape = apply(t1[c('t1','t2','t3','t4',

't5')],1,max)/t1$l.t.width.std

t.shape = replace(t.shape, is.nan(t.shape), 0)

#NoW WE CREATE A DATASET WITH oUR VARIABLES FoR THE oRDINATIoN

data.ord = data.frame(largest.t.pos=Dataset2$lm.largest.t.pos.res,

variance.teeth,min.max,Li.Lo=Dataset2$Li.Lo,

Lo.Li=Dataset2$Lo.Li,pos.bet)

rownames(data.ord)=Dataset2$Species

write.csv(data.ord,'data.macs.csv')

data.ord2 = data.frame(largest.t.pos2=t.pos.to.jl,

variance.teeth,min.max,Li.Lo=Dataset2$Li.Lo,

Lo.Li=Dataset2$Lo.Li,pos.bet)

rownames(data.ord2)=Dataset2$Species

write.csv(data.ord2,'data.macs2.csv')

#=============================================

**Code for Phylogenetic Least Squares Analysis (PGLS)**

rm(list=ls()) #clears R workspace (helps prevent errors from old data)

#-------------------------------------------------------------------------------------

setwd('C://Users//Ejer//Desktop//PhD//Fish Teeth. Morphology and Function')

library(phytools)

library(ape)

library(vegan)

library(dplyr)

library(ape)

library(geiger)

library(nlme)

library(phytools)

Dataset2 = read.csv('Dataset2.csv')

Dataset2 = Dataset2 %>% group_by(Species) %>%

summarise_all(mean,na.rm=T)%>% na.omit

tr=read.tree(file='tr.tre')

tr$tip.label = gsub("_"," ",tr$tip.label)

Morphotypes = read.csv('Envar1.csv')

data.ord = read.csv('data.ord.csv')

data.ord = data.ord %>% mutate(Types=Morphotypes$Type) %>%

mutate(Species = Morphotypes$Species)

rownames(data.ord) = data.ord$Species

rownames(data.ord)=gsub("Oxycheilinus digrammus","Oxycheilinus digramma",

rownames(data.ord))

data.ord$Species=gsub("Oxycheilinus digrammus","Oxycheilinus digramma",

data.ord$Species)

func.ord = read.csv('func.ord.csv')

#make datasets have the same order names as tr

tiplabels = as.vector(tr$tip.label)

data.ord = data.ord [match (tiplabels, data.ord$Species),]

rownames (data.ord)= data.ord$Species

tr = multi2di(tr)

rownames(Dataset2)= Dataset2$Species

Dataset2 = Dataset2[match(tiplabels, Dataset2$Species),]

func.ord = func.ord[match(tiplabels, func.ord$X),]

#but need to do the same for Morphotypes as well

Morphotypes = Morphotypes[match(tiplabels,Morphotypes$Species),]

#----------------------PGLS Brownian vs. Pagel-----------------------------------

library(AICcmodavg)

Type = Morphotypes$Type %>% as.factor()

a = func.ord$Dataset.std.res.a

c = func.ord$Dataset.std.res.c

prot = func.ord$Dataset.std.res.prot

mouth.shape = func.ord$mouth.shape

Li.Lo = func.ord$raw..Li.Lo.

Lo.Li = func.ord$raw..Lo.Li.

PGLS = list()

PGLS[[1]] = gls(Li.Lo~Type,

correlation = corBrownian(phy = tr),data = func.ord,

method = "ML")

PGLS[[2]] = gls(Li.Lo~Type,

correlation = corPagel(1,phy = tr,fixed = F),data = func.ord,

method = "ML")

AIC (PGLS[[1]], PGLS[[2]])

#---------------------------------------------------------------

PGLS[[3]] = gls(prot2~Type2,

correlation = corBrownian(phy = tr2),

method = "ML")

PGLS[[4]] = gls(prot2~Type2,

correlation = corPagel(1,phy = tr2,fixed = F),

method = "ML")

AIC (PGLS[[3]], PGLS[[4]])

#-----

PGLS[[5]] = gls(Type~SL*prot,

correlation = corBrownian(phy = tr),data = Dataset2,

method = "ML")

PGLS[[6]] = gls(Type~SL*prot,

correlation = corPagel(1,phy = tr,fixed = F),data = Dataset2,

method = "ML")

AIC (PGLS[[5]], PGLS[[6]])

#-----

PGLS[[7]] = gls(Type~c*prot,

correlation = corBrownian(phy = tr),data = Dataset2,

method = "ML")

PGLS[[8]] = gls(Type~c*prot,

correlation = corPagel(0,phy = tr,fixed = T),data = Dataset2,

method = "ML")

AIC (PGLS[[7]], PGLS[[8]])
